# A short educational intervention diminishes causal illusions and specific paranormal beliefs in undergraduates

**DOI:** 10.1371/journal.pone.0191907

**Published:** 2018-01-31

**Authors:** Itxaso Barberia, Elisabet Tubau, Helena Matute, Javier Rodríguez-Ferreiro

**Affiliations:** 1 Departament de Cognició, Desenvolupament y Psicologia de la Educació, Universitat de Barcelona, Barcelona, Spain; 2 Institut de Neurociències, Universitat de Barcelona, Barcelona, Spain; 3 Departamento de Fundamentos y Métodos de la Psicología, Universidad de Deusto, Bilbao, Spain; Universidad de Granada, SPAIN

## Abstract

Cognitive biases such as causal illusions have been related to paranormal and pseudoscientific beliefs and, thus, pose a real threat to the development of adequate critical thinking abilities. We aimed to reduce causal illusions in undergraduates by means of an educational intervention combining training-in-bias and training-in-rules techniques. First, participants directly experienced situations that tend to induce the Barnum effect and the confirmation bias. Thereafter, these effects were explained and examples of their influence over everyday life were provided. Compared to a control group, participants who received the intervention showed diminished causal illusions in a contingency learning task and a decrease in the precognition dimension of a paranormal belief scale. Overall, results suggest that evidence-based educational interventions like the one presented here could be used to significantly improve critical thinking skills in our students.

## Introduction

The development of successful debiasing strategies has been argued to be one of the most relevant contributions that Psychology could make to humanity [1]. Debiasing techniques are aimed to eliminate or, at least, diminish the frequency or intensity of the cognitive biases that populate our reasoning [1]. Everyday tasks are commonly based on heuristic processes or mental shortcuts that enable fast and computationally low demanding decisions. However, these heuristics sometimes produce cognitive biases, that is, systematic errors that distance us from normative reasoning and lead us to erroneous conclusions and suboptimal decisions [2].

Cognitive biases have been specifically related to various threats to human welfare including the acquisition and persistence of superstitious and pseudoscientific beliefs [3–5]; the emergence of group stereotypes and prejudices [6]; ideological extremism [1]; medical diagnostic errors [7,8]; or spurious therapeutic effectiveness [9]. Furthermore, they might also contribute to psychopathological conditions such as social phobia [10], depression [11], eating disorders [12] or to the development of psychotic-like experiences in healthy adults [13].

The extensive literature investigating the dangers posed by cognitive biases has encouraged research aimed to determine the circumstances under which these biases develop. It has been shown that situations which promote analytical thinking, such as the use of difficult-to-read fonts [14,15] or presenting information in a foreign language [16,17], diminish the effects of cognitive biases. Nevertheless, specific evidence-based interventions for debiasing that can be implemented as educational tools are still sparse.

Overcoming cognitive biases is not trivial because these biases often defy common sense and require to put our intuitions into question [9]. Furthermore, debiasing efforts usually find resistance because people do not like being exposed to their own flaws and the advantages of normative strategies are not obvious to them [2]. Examples of recent successful debiasing interventions include perspective taking techniques, which have been shown to produce durable reductions of intergroup prejudices [18], and probability training, which has been shown to yield positive effects to very complex reasoning activities such as geopolitical forecasting [19].

Promising results have also been observed in relation to interventions aimed to reduce causal illusions [20], which will be the main focus of this paper. Causal illusions, or illusions of causality, refer to the erroneous perception of a causal relationship between two events when no such causal relationship exists [5,21–24] (note that we also include what previous literature has sometimes referred to as “illusion of control” under the broader term “causal illusion” or “illusion of causality”). It has been suggested that this bias could be an important contributing factor to the development and maintenance of superstitious and pseudoscientific beliefs [5,21,25]. Causal illusions are typically studied in the laboratory by means of a standard contingency learning task [26–28]. In this task participants are asked to evaluate a potential causal relationship between two events, for example the effectiveness of a new drug, the potential cause, for curing a fictitious disease, the outcome of interest [21]. With this goal in mind, participants are typically presented with medical records from several fictitious patients, presented one by one, that either took the drug or not, and they observe whether each patient recovered from the fictitious disease or not. Importantly, when the situation is set up by the experimenters so that the patients are healed irrespective of the administration of the drug or not (i.e., the probability of healing is equal among patients taking and not taking the drug), sometimes participants incorrectly conclude that the drug is producing the occurrence of the outcome [21]. This is known as a causal illusion because participants illusorily perceive the drug (the potential cause) as causing the recovery of the patients (the outcome). This illusion is facilitated when the probability of the outcome is high (outcome density effect, e.g. [26,29,30]), and when the probability of the potential cause is high (cue density effect, e.g. [5,23,29]), leading to particularly intense causal illusions when both probabilities are high [21,31]. Moreover, it has been shown that in situations where the percentage of healings is high and participants are allowed to choose between giving or not giving the drug, they are inclined to administer the drug to a majority of the patients, thereby tending to expose themselves to more patients that take the drug than to patients that do not take it [20,32]. The presence of this spontaneous search strategy is especially relevant because, as we have already noted, the increase of the percentage of trials in which the potential cause is present fuels the intensity of the causal illusion that they develop.

In everyday life, the situations where miracle pills and unproven therapies are perceived to be successful can be linked to circumstances that facilitate the emergence of causal illusions. These ineffective products and therapies are usually applied to conditions with high rates of spontaneous remission, such as, for instance, back pain [33]. As we have already explained, high rates of the desired outcome (i.e., a high probability of spontaneous improvement or relief from the illness) increase the tendency of the user to develop causal illusions (i.e., the erroneous perception of the product being effective). The illusory perception of efficacy, in turn, can foster the use of the product and hence strengthen false beliefs that are propagated among others who end up sharing the illusion.

With this in mind, Barberia et al. [20] conducted a study with adolescents. Volunteers in the intervention condition participated in a workshop in which they were offered direct experience with a bogus miracle product. After being fooled that the product had improved their physical and cognitive abilities in different tasks, the participants were debriefed and they received a tutorial on experimental methods including advice on how to reliably establish causality. Compared to a control group who had not received the intervention, participants in the intervention group showed a weaker causal illusion in a standardized contingency learning task. Moreover, the authors suggested that the decrease in the illusion could be, at least in part, due to a change in the behavior of the participants that had received the intervention, as they exposed themselves to less cause-present trials (they administered the drug to fewer patients and, accordingly, they could observe the outcome in more patients not taking the drug). Despite the evident value of these results, it could be argued that the intervention and measure procedures were too aligned, what casts doubt on the transferability of the acquired knowledge. Moreover, it remains unclear whether the effects of the intervention would extend to more general beliefs that seem to be associated with causal illusions such as paranormal beliefs [25].

In the current study we present a new example of a successful educational intervention aimed to reduce the impact of cognitive biases on causal reasoning as well as to encourage a more critical analysis of paranormal beliefs. Our present intervention was specifically designed to overcome two problems that have been noted to undermine the success of debiasing interventions [1]: the “bias blind spot”, which refers to the tendency to not accept that one’s perspective might be biased while being able to recognize biases on the judgment of others [34,35], and the lack of perceived personal relevance of the cognitive biases [36,37]. To this respect, we started the intervention with a staging phase that induced cognitive biases in our participants so as to demonstrate how easily we can all be tricked to commit these thinking errors. Thereafter, we provided various examples of everyday situations in which the presented biases play a role in order to illustrate the extent to which cognitive illusions are important to our daily lives.

Our debiasing techniques can be situated among cognitive strategies [2]. In this sense, we applied a training-in-bias approach [2] focusing on two important cognitive phenomena, namely the Barnum effect and the confirmatory strategy elicited by the 2-4-6 task. The Barnum or Forer effect [38,39] refers to the tendency to accept and rate as highly accurate vague personality descriptions that are presented as specific and personalized but are actually so common that they can be applied to almost anyone. We considered that the Barnum effect would be strongly and easily induced in most of the participants, what would help overcoming the “bias blind spot”, and that inducing this effect was also appropriate in order to enhance the perceived personal relevance of cognitive biases, as it is easily applied to everyday situations. On the other hand, the 2-4-6 task has been shown to elicit a confirmatory searching strategy [40,41]. We considered that presenting this task was especially relevant because, as previously described, biased information search has been proposed to play a role in causal illusions [20]. Specifically, when participants are presented with a potential causal relationship in the contingency learning task, they tend to test this relationship by choosing to preferentially observe cases in which the potential cause is present, what can be considered a confirmatory search strategy.

Given that the mere awareness that a cognitive flaw exists is not enough for overcoming its effects [2], our intervention was complemented with a training-in-rules methodology focused on pointing out the "consider the opposite" approach. In situations where a person is required to make a judgment, this strategy consists of searching for possible reasons why an initial consideration or hypothesis might be wrong as an effective way to diminish confirmatory tendencies by favoring discovery and evaluation of new information [2].

We conducted our study with groups of Psychology undergraduates. The effect of the intervention over causal illusions was assessed by means of a standardized contingency learning task. Moreover, we added a measure of paranormal beliefs in order to investigate the generalizability of the observed effects to different domains of superstition. A previous study found that causal illusions generated in a contingency learning task tend to correlate with some types of paranormal beliefs [25]. If, in line with previous results [20], our debiasing intervention were able to diminish causal illusions, we could speculate that it might also impact these correlated beliefs. In sum, we expected our intervention to influence the learning strategies of our students and their causal judgments, promoting a more critical approach to the discovery of new information and reconsidering of a priori beliefs.

## Methods

### Participants

A total of 106 Psychology undergraduates took part in the study (86 females). Forty-seven students (mean age 21.57, SD 3.48, 36 females) received the intervention condition and 59 students received the control condition (mean age 20.83, SD 2.65, 50 females).

The study was performed into a regular class of the Psychology degree, in the context of a teaching initiative aimed to promote scientific thinking among students. Importantly, prior to the intervention participants were only informed that the initiative aimed to promote transversal competences, but not that it was specifically addressed to practice scientific thinking. All students that attended the class participated in the intervention and its assessment. However, students could decide, at the end of the class session, if they wanted to consent for their data to be used anonymously for research purposes or not. Only the data from students that gave written consent are presented. The study, which complied with APA ethical standards, was approved by the ethics committee of the University of Barcelona (Comissió de Bioètica de la Universitat de Barcelona).

### Procedure

The intervention and assessment (see below) were carried out in a 90 min session included into regular courses of the Psychology degree. We conducted three experimental sessions with three different groups of students. Participants in each session were randomly distributed to two different rooms, corresponding to the intervention or control conditions, respectively. The rooms were equipped with one desktop computer per student. The students in the intervention condition received the educational intervention before assessment of their causal illusion and paranormal beliefs, whereas, for the students in the control condition, the assessment was carried out first, and then, due to ethical considerations, the intervention was also provided.

The same instructor conducted the intervention condition across the three sessions. Simultaneously, other instructors conducted the control condition in the other room. Note that differences due to the involvement of different instructors in the intervention and control conditions cannot be expected to influence our results because the assessment in the control group was presented before any intervention, and the instructions for the assessment tasks were provided in written form for both intervention and control conditions.

### Intervention

The educational intervention consisted of a staging phase followed by a debriefing phase. The staging phase started with the bogus explanation of a psychological theory according to which a fine-grained personality description can be obtained from the analysis of performance in low-level cognitive tasks. Then the participants were asked to carry out two computer tasks related to this theory. We explicitly prompted students to work individually during the tasks focusing on their own computer screens. The initial screen requested participants to state their age and gender. The first task, inspired by an on-line quiz (http://braintest.sommer-sommer.com), was presented as a personality assessment and consisted of a point-and-click version of the Stroop test as well as a pattern selection test in which the participant simply had to choose which of three different arrangements of colored geometrical figures was most similar to a given target. After completing these two simple tests the computer supposedly analyzed the data and provided an allegedly individualized personality description. The report consisted of an adaptation of most of the original sentences used by Forer [38], although the order of the sentences was randomized for each participant in order to hinder identification of the hoax in case the students could see other participant’s description. The descriptions were gender-adapted in order to increase the degree of perceived personalization of the description. After they read their personal report, the participants were asked to indicate in a 0 to 100 scale “to what extent you think the test has been effective detecting how you are”.

The second task of the staging phase of the intervention was presented as a test of reasoning abilities and was a computerized version of the 2-4-6 task [40] adapted from http://www.devpsy.org/teaching/method/confirmation_bias.html. Participants were asked to identify a rule that applied to triplets of numbers. They were first given the sequence 2-4-6 as an example of a triplet that satisfied the rule. Then the volunteers had the opportunity to generate new triplets to test whether they followed the rule or not. After they introduced each triplet the computer provided feedback about the triplet fitting the rule or not. Participants could continue testing triplets until they were sure of the exact rule (they could test a maximum of 20 triplets). After each triplet-testing trial the participants were asked to declare their rule in mind together with their confidence in the correctness of their hypothesized rule. The participants were, hence, free to test different rules throughout the task. However, they were not told whether their rule was correct or not until the debriefing phase of the intervention. In this task, participants typically form a specific hypothesis about the rule such as “numbers increasing by twos” and then tend to generate triplets that follow the rule they are testing. This positive testing strategy [41,42] is ineffective in this specific task because the original rule is more general (i.e. “increasing numbers”). Alternatively, a “consider the opposite” strategy, here testing examples that do not satisfy the rule, leads to the formation of new, broader, hypotheses, and eventually to the discovery of the correct one (note that we assume along the paper that the positive testing strategy involves a confirmation bias, but see [41] and [42] for a debate on this).

The debriefing phase of the intervention started after all the participants had finished the two tasks. In this phase, we provided theoretical explanations of the Barnum effect and of the typical performance in the 2-4-6 task. We first introduced the original study by Forer [38] together with the personality description used by him. At this point the students realized that it was the same description they had received, and we informed them that the initial theory and the personality test were fake. We then discussed the results found by Forer in his study and the students were free to intervene giving their impressions. After, we moved to the Wason [40] study, and illustrated the students with both the typical confirmatory strategy used in the 2-4-6 task and with the more effective “consider-the-opposite” strategy (examples taken from http://www.devpsy.org/teaching/method/confirmation_bias.html). This was completed with a description of the confirmation bias, defined as the tendency to partially search, select or interpret confirmatory information that leads to the acceptance of a priori beliefs or expectations while ignoring alternative information that could lead to their rejection [41]. Finally, we explained how these cognitive biases are involved in situations like reading your horoscope in a magazine or taking a graphological assessment, as well as false beliefs like the full moon effect [43], or questionable effects such as the alleged relation between articular pain and relative humidity [44].

### Assessment

The assessment phase consisted of two different parts. First the participants completed a contingency learning task, and second they answered a paranormal beliefs questionnaire.

As we have already explained, in a standard contingency learning task participants are asked to assess a potential causal relation, in our case between taking a drug and relieving from a disease. Our volunteers performed a computer task in which they were asked to take the role of a medical doctor whose goal was to determine whether a given drug was effective or not. They were sequentially presented with 40 fictitious cases of patients that suffered a fictitious disease. In each trial they had the opportunity to administer the drug to the patient. Then the participants were informed whether the patient was healed or not. The healings occurred following a pre-programmed randomized sequence, so that 6 out of every 8 patients were cured, both among the fictitious patients receiving the drug and among those that did not receive it. That is, the drug did not increase the probability of healing and was therefore ineffective. The rate of relief was programmed to be high (.75), in order to simulate a condition that promotes the development of causal illusions [21,32]. The anticipated default strategy (i.e., the one expected in the participants from the control group) would involve administrating the drug frequently and, as a consequence, being exposed to more cause-present than cause-absent trials, hence developing a causal illusion, as has been shown in previous studies [20]. Once participants had gone through the full set of patients, they were asked to evaluate the effectiveness of the potential cause (the drug) producing the outcome of interest (healings) on a scale ranging from 0 (not effective at all) to 100 (totally effective). This judgment of causality was our main dependent variable. Given that the relationship was, in fact, inexistent, higher judgments were interpreted as a stronger causal illusion formed by the participant.

Regarding paranormal beliefs, we used the Spanish adaptation [45] of the Revised Paranormal Beliefs Scale [46,47] which consists of 30 items answered in a Likert scale from 1 (“totally disagree”) to 7 (“totally agree”). The scale provides a global score of paranormal beliefs as well as a score in eight different subscales (see Table 1 in reference [45] for the items that we included in each subscale): witchcraft, psi, traditional religious beliefs, spiritualism, extraterrestrial life and actual visits, precognition, superstition and extraordinary life forms. This version of the scale has been standardized with a sample of undergraduate students and shows large reliability scores (Cronbach’s alpha 0.91) [45]. Following [45], item 23 was not included in the calculation of our scores. Note, also, that we substituted the wording of item 20, "There is life on other planets", by "There is intelligent life on other planets". When a participant failed to answer to a specific item, her score (either the global score or that of any subscale) was calculated by averaging the rest of the items.

## Results

The statistical analyses were performed using JASP [48]. We performed Bayesian t-tests using JASP’s default Cauchy prior width, *r* = 0.707. We interpreted Bayes factors following Table 1 in reference [49]. We constructed the plots by means of the YaRrr! package [50] in R [51]. The dataset is available at https://osf.io/vq5b7/.

Before we analyze the effectiveness of the intervention, it is worth looking at the results of the Barnum task. This activity was performed in both conditions at the beginning of the intervention, therefore its results cannot be used as a measure of the effectiveness of the intervention. However, the results are informative of the degree to which the Barnum effect was present in our sample. In a 0 to 100 scale our participants evaluated the accuracy of the bogus description with a mean rating of 83.85 points (*SD* = 12.77) in the intervention group and a mean rating of 78.62 points (*SD* = 20.62) in the control group. As expected, the effect of condition (intervention vs. control) was not significant, *t*(103) = 1.518, *p* = .132, *d* = 0.298. A two-sided Bayesian independent samples t-test (intervention ≠ control) suggested anecdotal evidence for the null hypothesis, BF_10_ = 0.577.

**Fig 1:**
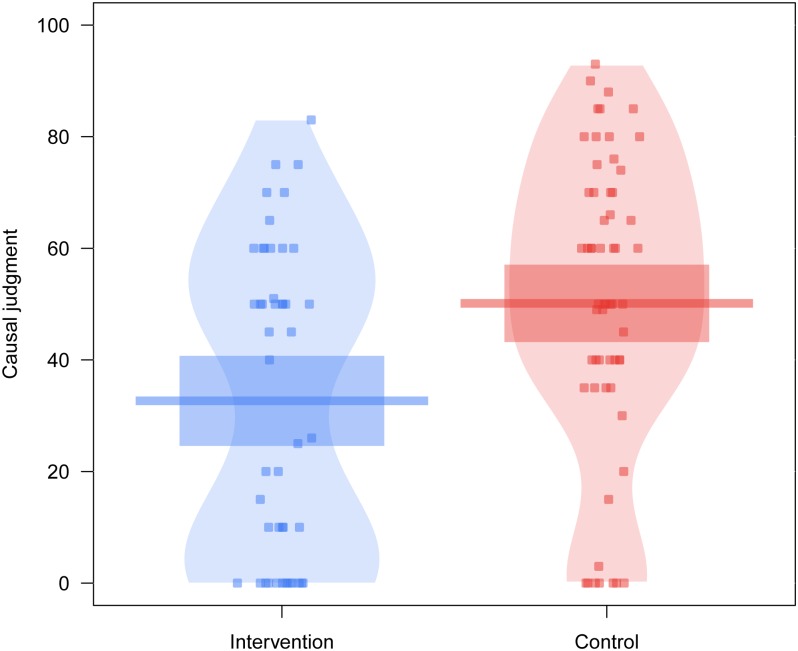
Intensity of the causal illusion. For each condition, the points represent the raw data, the horizontal lines represent the mean causal judgments, and the rectangles the 95% confidence intervals.

Fig 1 shows the results of the contingency learning task used to measure the amount of causal illusions developed by the participants. As can be seen, participants in the intervention group developed a weaker causal illusion, as shown in their causal judgments being closer to zero than those of the control group. A one-sided t-test for independent samples (intervention < control) over the causal judgments showed a significant effect of the intervention, *t*(104) = -3.313, *p* < .001, *d* = -0.648. A one-sided Bayesian independent samples t-test suggested very strong evidence in favor of the alternative hypothesis, with a Bayes factor of BF_10_ = 47.69. This indicates that our results are 47.69 times more likely under the hypothesis that ratings in the intervention group are lower than those in the control group.

**Fig 2:**
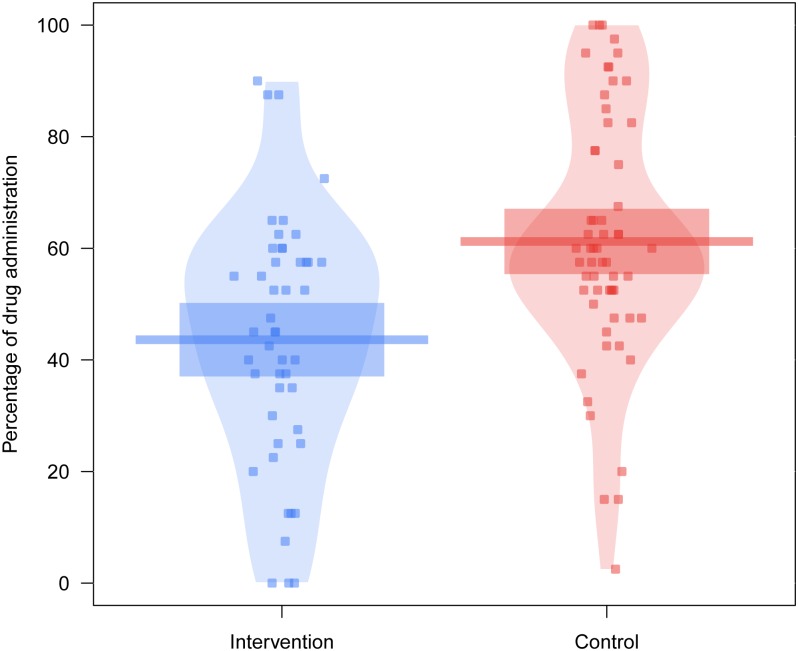
Percentages of drug administration. For each condition, the points represent the raw data, the horizontal lines represent the mean percentage of drug administration, and the rectangles the 95% confidence intervals.

Fig 2 summarizes the participants’ search strategy during the contingency learning task. Specifically, it shows the percentage of trials in which participants decided to administer the fictitious drug to the patients, that is, the percentage of cause-present trials they exposed themselves to. As anticipated, participants in the control condition adopted the expected default strategy (i.e. high drug administration rate), as they gave the drug to more than 50% of the patients, one-sided t-test *t*(58) = 3.840, *p* < .001, *d* = 0.500. This strategy was not shown by the participants in the intervention condition, *t*(46) = -1.952, *p* = .971, *d* = -0.285. The Bayesian analogue analysis indicated extreme evidence favoring the hypothesis that participants’ percentage of drug administration was higher than 50%, BF_10_ = 157.1 in the control group. In contrast, in the intervention group there was strong evidence favoring the hypothesis that participants did not administer the drug more than 50% of the time, BF_10_ = 0.057.

Furthermore, a one-sided t-test for independent samples over the percentage of trials in which participants administered the drug confirmed the hypothesis that participants in the intervention condition administered the drug less frequently than those in the control condition, *t*(104) = -4.014, *p* < .001, *d* = -0.785. The corresponding Bayesian analysis showed extreme evidence in favor of this hypothesis, BF_10_ = 395.2.

Previous studies have shown that manipulating the probability of the potential cause, in our case, the proportion of cases in which the drug was administered, impacts the intensity of causal illusions. Specifically, the higher the proportion of cause-present trials the stronger the causal illusion developed [5,23,29]. Since the intervention and control groups differed in the percentage of drug administration, it is plausible to assume that differences in the strength of the causal illusion between groups might be predicted by this variable. With this in mind, we performed a regression analysis in order to state the extent to which the effects of our intervention over causal judgment could be associated to differences in drug administration rates. Moreover, following the suggestion of a reviewer, we also decided to introduce the experienced contingency as an extra predictor in the analysis. Given that participants could decide in which trials they wanted to administer the medicine or not, the actual contingency experienced by each participant, defined as the difference between the probability of the outcome in the presence and absence of the potential cause [52], could depart from the programed contingency of zero. We, thus, conducted a regression analysis including condition (intervention, control), percentage of drug administration, and experienced contingency as independent variables and causal judgments as dependent variable. Results showed a significant effect of percentage of drug administration (β = .653, *p* < .001) but no significant effect of condition (β = .064, *p* = .413), neither of experienced contingency (β = .022, *p* = .784). These results suggest that the intervention might have impacted causal judgments by decreasing the tendency of the participants to administer the drug.

Regarding our measure of paranormal beliefs, a one-sided independent samples t-test (intervention < control) showed no significant effect of the intervention in the global scores of the Revised Paranormal Beliefs Scale (intervention: mean 2.26, SD 1.01; control: mean 2.33, SD .95, *t*(102) = -0.396, *p* = .346, *d* = -0.078). The Bayesian version of the analysis showed moderate evidence favoring the null hypothesis, BF_10_ = 0.288. Separate one-sided analyses of the scores corresponding to the different test subscales (intervention < control) showed a significant effect of the intervention in the precognition subscale, *t*(102) = -2.616, *p* = .005, *d* = -0.515, an effect that survived Bonferroni correction for multiple comparisons (adjusted *α* = .006). None of the other seven subscales reached the significance threshold (*ps* > .26). Accordingly, a one-sided Bayesian independent samples t-test analysis returned a Bayes factor of BF_10_ = 8.247 for the Precognition subscale (which can be considered moderate evidence favoring the alternative hypothesis that the intervention group presented lower Precognition scores than the control group). In contrast, the Bayes Factors (BF_10_) for the rest of the subscales suggested anecdotal to moderate evidence in favor of the null hypothesis (Witchcraft 0.338; Psi 0.284; Traditional religious beliefs 0.178; Spiritualism 0.181; Extraterrestrial life and actual visits 0.184; Superstition 0.361; Extraordinary life forms 0.271).

## Discussion

The goal of this study was to develop a debiasing intervention aimed to diminish the influence of cognitive biases over everyday reasoning and to promote a critical perspective in relation to pseudoscientific and superstitious beliefs. We conducted our intervention with Psychology undergraduates, who showed a classic Barnum effect with a mean description acceptance rating over 80 points out of 100. We thus replicated the results obtained in the original experiment by Forer [38] who registered a mean rating of 4.3 out of 5. These results suggest that even higher education students are susceptible to accept pseudoscientific claims [4,53]. As we have already noted in the Introduction, we decided to use causal illusions as the main measure for this study because biases affecting causal inference are assumed to be at the core of pseudoscience and superstition [5]. Barberia et al. [20] observed a reduction of causal illusions in volunteers that had been specifically trained in the rationale of scientific inferences about causal relations, focusing on the concept of contingency and the need for appropriate control conditions. In the present study, we aimed to test whether a more general approach without explicit training in causal relation identification could yield a similar effect.

We combined training-in-bias and training-in-rules techniques by evoking two well-known cognitive biases in the volunteers and explaining how they influence our judgments and/or decisions in relation to different topics. This procedure allowed us to point out how easily cognitive illusions can be elicited and raise awareness on their relevance for everyday life, thus addressing known threats to debiasing interventions such as the bias blind spot [34,35] and the lack of perceived personal relevance [36,37]. Furthermore, it also provided the opportunity to introduce the volunteers to the general idea of maximizing the availability of information before a given decision situation by means of “consider the opposite” strategies [2].

Our intervention decreased the illusion of causality as evidenced by the lower causal ratings provided by the intervention group in the contingency learning task in comparison to the control group. Moreover, the results of the regression analysis indicate that the reduction of the causal illusion could be mainly attributable to a decrease of the exposure to the potential cause and, accordingly, to an increment in the chances to observe the outcome during the, now more frequent, cause-absent trials. That is to say that volunteers in the intervention group might have developed the causal illusion to a lesser extent because they tended to generate more cause-absent trials than participants in the control group. We argue that this approach results from the application of a general disconfirmatory or “consider the opposite” strategy presented in the intervention to a specific causal context. During the explanation of the 2-4-6 task we pointed out that in this context a positive testing strategy is unsuccessful whereas testing examples that do not follow the initial rule may lead to the consideration of new hypotheses and, finally, the discovery of the correct rule [41,42]. In our contingency learning task, generating cases in which the cause is present by giving the drug to the patient is analogous to the positive testing strategy used in the 2-4-6 task because it involves a preference to search for cases in which the outcome is expected to occur if the initial hypothesis (i.e. “the drug is effective”) were true. Conversely, the generation of cause-absent trials is equivalent to testing triplets that do not follow the hypothesized rule because it implies searching for examples where the outcome is expected not to occur in case the drug is responsible for healing.

Finally, we also included a questionnaire of paranormal beliefs in order to test whether the effect of our intervention extended to the participants’ credences in relation to these beliefs. Our analyses showed that overall scores were unaffected by the treatment. However, the results showed moderate evidence suggesting that the intervention could specifically impact scores of one of the subscales of the questionnaire, the Precognition subscale. This subscale refers to abilities to predict the future via paranormal means and it is comprised of items referring to horoscope and astrology among other topics. In our intervention, horoscope appeared as an example aimed to illustrate the influence of cognitive biases in our lives. Horoscope predictions of personality and future events usually rely on vague descriptions that can be applied to a wide range of people, a key aspect in the acceptance rates of Barnum-like descriptions [54]. Moreover, these descriptions tend to include high proportions of favorable statements, eliciting confirmation bias-related phenomena such as the self-enhancement effect [55]. The fact that we explicitly mentioned this kind of examples might have been responsible of the observed result in relation to the precognition subscale. Nevertheless, the effect of our intervention failed to generalize to other dimensions of paranormal belief that were not directly addressed during the intervention.

One limitation of this study is that our results rely exclusively on between-participants comparisons. In this sense, although students were randomly assigned to one of the two conditions, we cannot totally rule out initial differences between participants in the control and intervention groups. This limitation could be overcome in future research by carefully designing studies that allow collecting pre- and post-intervention measures from the same participant.

A second limitation relates to the complex nature of our intervention, comprising the direct experience and subsequent explanation of both the Barnum effect and the confirmation bias in relation to the 2-4-6 task, as well as the discussion on the potential implications of these effects on everyday life. With our design we cannot disentangle which, if not all, of the components of the intervention are responsible for its beneficial effects. Future designs isolating each of these components could shed light on this issue and potentially contribute to the design of more efficient interventions.

In conclusion, with this study we move forward in the direction started by previous research aimed to provide evidence-based educational tools to overcome the detrimental effects of cognitive biases. Our results suggest that an evidence-based educational intervention such as the one we present here could be used to significantly improve scientific thinking skills in adults, decreasing their probability of developing causal illusions that can be on the basis of several misbeliefs.
